# COVID-19 & Sociocultural Determinants of Global Sanitation: An Aide-Mémoire and Call to Decolonize Global Sanitation Research & Practice

**DOI:** 10.5334/aogh.3358

**Published:** 2021-09-14

**Authors:** Ans Irfan, Denise T. St. Jean

**Affiliations:** 1 Harvard Divinity School, Cambridge, MA, US; 2 Department of Environmental & Occupational Health, Milken Institute School of Public Health, George Washington University, DC, US; 3 Department of Epidemiology, Gillings School of Global Public Health, University of North Carolina at Chapel Hill, Chapel Hill, NC, USA

## Abstract

COVID-19 has highlighted and exacerbated many global health inequities. Emerging evidence suggests that SARS-CoV-2 can spread through fecal aerosols, making sanitation a critical part of the COVID-19 mitigation strategy and providing an opportunity to reflect on current challenges and opportunities related to global sanitation at large. Global sanitation interventions continue to fall short of their target expectations, leading to millions of deaths and illnesses worldwide. Eurocentric approaches to sanitation fail to account for sociocultural determinants of sanitation behaviors and health, leading to low sanitation intervention uptake. Global public health needs to take a decolonial approach to our research and practice, and meaningfully involve local communities to progress towards global health equity.

The coronavirus disease 2019 (COVID-19) pandemic has highlighted many longstanding, persistent global health inequities [1,2]. Severe acute respiratory syndrome coronavirus 2 (SARS-CoV-2), the virus underlying the COVID-19 pandemic, has affected the entire world at a scale not seen since the 1918 Spanish Flu pandemic over a century ago [3,4]. As of July 2021, there have been over 187 million cases and over 4 million deaths worldwide [5]. Socially vulnerable populations across the globe, including racial and ethnic minorities, the elderly, the poor, and people with the lowest educational attainment, have been most severely impacted by the pandemic [6,7,8]. SARS-CoV-2 spreads primarily through respiratory droplets; hence, preventative measures such as mask wearing, limitations on social gathering, and physical distancing mandates have been prioritized [9]. However, mounting evidence suggests that transmission may also occur through fecal aerosols [10,11,12,13,14,15,16]. Fecal aerosol [17] transmission of SARS-CoV-2 includes potential exposures through shared facilities—currently used by over 600 million people globally—where the virus has the potential to become airborne if utilized by a person with active infection [18]. According to a systematic review of over 90 studies including stool samples or anal swabs from COVID-19 positive patients, viral shedding of SARS-CoV-2 in stool samples occurred in over 40% of patients, and patients tested positive for a mean of 25 days after symptom onset [19]. This emerging body of evidence has put the global health community on alert and brought global sanitation—an otherwise relatively neglected area of global health praxis—into the limelight [20,21,22]. This has led to a heightened interest in global sanitation as a potential COVID-19 mitigation strategy. It also provides an opportunity to review the fundamentals of global sanitation and highlight challenges and opportunities in this sector, especially as they relate to Eurocentric practices that often overlook local sociocultural determinants of sanitation and inevitably leads to poor sanitation interventions.

Sanitation—an essential ingredient of human health and dignity—is the key to achieving global health equity through the Sustainable Development Goals. Sanitation is a critical component of water, sanitation, and hygiene (WASH) programming. WASH interventions attempt to reduce morbidity and mortality related to water, sanitation, and hygiene in settings such as refugee camps, schools, healthcare facilities, and humanitarian disaster settings in low- and middle- income countries (LMICs) [23]. Ongoing efforts to improve sanitation have consistently fallen short of achieving global sanitation goals [24]. At the end of 2020, 2 billion people still lacked even very basic sanitation services such as toilets or latrines [25]. Of these, nearly 700 million engage in open defecation [22]. Moreover, over half of the global population (4.2 billion people) still lack “safely managed sanitation” facilities, defined as access to a toilet or latrine leading to treatment or safe disposal of human excreta [22]. These alarming trends have led to an increase in many health and social ills ranging from gender-based violence to infant mortality [26]. Lack of sanitation disproportionally affects the most socially vulnerable populations such as poor communities, sexual and gender minorities, and children, making it a quintessential global health equity issue.

Inadequate sanitation, directly or indirectly, is responsible for significant health and economic losses in LMICs. Over 830,000 annual deaths due to diarrheal disease, and many more from other disease types, are attributable to inadequate sanitation [27]. Approximately 58% of deaths caused by diarrheal diseases—the key driver behind sanitation-related mortality—are attributable to poor sanitation and unsafe water [28]. Among children under five, diarrhea kills over half a million children—a sobering statistic, greater than AIDS, malaria, and measles deaths combined [24]. Poor sanitation is also linked to other health conditions such as malnutrition, helminth infections, trachoma, schistosomiasis, lymphatic filariasis, guinea worm disease, Buruli ulcer, and respiratory illness [24]. While COVID-19-specific studies have yet to be conducted, randomized controlled trials in Mali [29] and Bangladesh [30] have demonstrated that sanitation interventions that improve the poor environmental conditions that support transmission of respiratory pathogens can reduce the incidence of respiratory illness. Lack of sanitation also results in large-scale economic losses. Globally, an estimated $260 billion and up to 7% of the gross domestic product of LMICs is lost each year due to poor sanitation [31,32]. These economic losses detract from investment in social welfare programming and further exacerbate structural health and social inequities.

Annually, $20 billion is invested in sanitation-related interventions. These are highly cost-effective investments that, according to the World Health Organization, lead to a $5.50 return-on-investment for every dollar spent [33]. However, critical challenges remain in achieving global sanitation goals, as briefly highlighted in the health and economic impacts discussed above. In fact, few other global public health interventions face a similar lack of uptake and underutilization by the target population. Many sanitation interventions suffer from low uptake and outright failures resulting in a loss of millions of lives and billions of dollars each year [34,35,36]. Evidence suggests that up to 50% of sanitation interventions fail to bring sustainable population-level benefit within two to five years of implementation [37]. This highlights a critical failure in our approach to sanitation interventions, most of which heavily rely on engineering-based, technical solutions. These solutions might be efficacious in a controlled environment but are not effective when implemented under “real-world” conditions.

Contemporary colonial approaches that exclude relevant stakeholders at the formative and financing stages of sanitation intervention development and Eurocentric global public health that fails to account for sociocultural nuances of the target populations are key reasons for low uptake and failures of sanitation interventions [38,39,40]. For example, the Transition Management framework in sanitation [41] and the Sanitary Hamlet Program in Vietnam [42] are excellent case studies of the limitations of a solely Western approach to introducing sanitation interventions in LMICs. Culture, the driving force determining sociocultural norms and behaviors, is embedded in all aspects of people’s lives—sanitation is no exception. This persistent challenge stems from Western researchers developing and implementing sanitation interventions without a deep understanding of the local cultural traditions. This not only perpetuates colonial mindsets across the research community, but also leads to ineffective interventions, billions in economic losses, and millions of human lives lost.

Sanitation is a fundamental part of cultural practices, religion, and community traditions and should be approached through a sociocultural, decolonial lens grounded in community involvement and empowerment. There is an enormous sociocultural variation around the globe in sanitation-related perceptions, beliefs, and attitudes. Global diversity of attitudes and beliefs towards sanitation are often described by the Winblad’s faecophilia–faecophobia continuum—which suggests certain societies having mostly positive or mostly negative attitudes towards feces that determine uptake of sanitation interventions [43]. However, this framework fails to capture the localized nuances, complexities, and variations across and within societies. It is not possible to describe every sociocultural variation in sanitation-related beliefs worldwide. However, some of these variations include toilets as a taboo (Madagascar) [44], sharing a toilet with in-laws as impermissible (parts of Kenya and Zambia) [45], open defecation as a sign of strength, health, purity, and longevity (several parts of Bangladesh, Nepal, India) [46,47], and a broad application of human feces as fertilizers (China) [48]. Religious diversity also widely influences sociocultural beliefs and practices as they relate to sanitation interventions. For instance, traditional upper-class Hindus, the Brahmins, associate feces with impurity and untouchability. This belief system has consistently stymied sanitation interventions in rural India involving latrines in homes [49]. Muslims are religiously sanctioned to wash their genitals after defecation, rendering technologies such as dry sanitation inoperable in predominantly Muslim countries and refugee camps with majority Muslim refugee population [50]. In certain instances, the direction of newly constructed toilets facing Mecca, a holy place for the Muslim community, led to a complete boycott of an intervention by the local Muslim community [43]. These examples highlight the sociocultural variation regarding sanitation and the urgent need to look beyond technocratic solutions to this complex phenomenon.

This context is critical to keep in mind for the global public health community to acknowledge the challenges associated with Western hegemonic universalism, rooted in colonial ideas of White supremacy. Sanitation interventions are more likely to succeed by centering the sociocultural determinants of sanitation and “the shared set of (implicit and explicit) values, ideas, concepts, and rules of behaviour that allow a social group to function and perpetuate itself [51].” We offer a few evidence-based strategies for achieving this monumental task of taking a decolonial approach towards global sanitation regime below.

## Infusing Qualitative Methods into Sanitation Programs & Policies [52,53]

Sanitation research and practice should focus on incorporating qualitative methods as a critical part of intervention development. Taking a mixed-methods approach that emphasizes qualitative methods can provide critical insights into community preferences ranging from latrine designs to local traditions and facilitate effective sanitation programming. More importantly, deeply understanding community context is key to the success of sanitation interventions and can only be done by integrating qualitative methodology in formative, process, and outcome evaluation of sanitation interventions.

## Centering Community-based Participatory Research (CBPR) [54,55,56]

CBPR principles should be incorporated into all aspects of sanitation research and programming. This will ensure local stakeholders and community leaders—especially the socially vulnerable and most affected groups such as women—are engaged in a meaningful way, leading to equitable development of effective interventions with a much higher potential for uptake. Moreover, by equitably involving relevant community stakeholders and ensuring their expertise is valued as much as that of academic researchers, interventions can be enriched exponentially while making progress towards a decolonial practice of global public health with shared ownership and decision-making.

## Engaging Faith Leaders [57,58]

As part of the CBPR, it is critical to engage spiritual, faith, and religious leaders for effective implementation and uptake of sanitation interventions. As trusted members of the local communities, these leaders can provide the necessary social capital and rally support for sanitation interventions to make them more acceptable to the community; hence, enabling potentially higher uptake of the sanitation interventions.

## Conclusion

In conclusion, sanitation research and practice community can play a significant role in advancing global health equity through culturally sensitive interventions that account for sociocultural determinants of sanitation practice. For effective COVID-19 mitigation and equitable sanitation programming in a post-COVID-19 world, it is imperative to understand local cultural context, meaningfully engage local communities across the intervention lifecycle, and integrate sociocultural determinants of sanitation in research, policies, programs, and practices, despite the hyperlocal nature and diversity of sanitation. As we look to reevaluate our approaches to global public health, including sanitation, this is an excellent opportunity to reflect and move forward with an intentionally decolonial research and practice that is rooted in global health equity, fairness, and justice.
